# Painful fat necrosis resulting from insulin injections

**DOI:** 10.1530/EDM-14-0073

**Published:** 2014-09-01

**Authors:** P Hanson, M Pandit, V Menon, S Roberts, T M Barber

**Affiliations:** 1 Endocrinology, University Hospital of Coventry and Warwickshire, Coventry, UK

## Abstract

The case is a 34-year-old woman with long-standing type 1 diabetes mellitus with existing follow-up in the outpatient clinic at the Warwickshire Institute for the Study of Diabetes, Endocrinology and Metabolism, UHCW. She had maintained good glycaemic control and glycaemic stability with basal bolus regimen for many years. She had not developed any diabetes-related complications and had no other co-morbidities. Six months ago, she presented to A&E with sudden-onset, well-localised and severe pain in the right iliac fossa, just lateral to the para-umbilical area. Her biochemistry was normal. Ultrasound scan, however, revealed a right-sided ovarian cyst, which was thought to have caused pain to her. She was discharged from A&E with simple analgesia. On subsequent gynaecological follow-up 4 weeks later, her pain remained severe and examination revealed an exquisitely tender subcutaneous nodule at the same location measuring 2 cm in diameter. Magnetic resonance imaging (MRI) scan at the time revealed a 1 cm mass in the subcutaneous adipose tissue, which co-localised to her pain. The mass demonstrated a central fat signal surrounded by a peripheral ring: observations consistent with fat necrosis. There were other smaller subcutaneous nodules also observed in the left para-umbilical area. Subsequent surgical resection of the main area of fat necrosis was performed. The patient made an excellent recovery and her pain resolved post-operatively. Histology confirmed the presence of fat necrosis. Fat necrosis is a rare complication of s.c. insulin injection. This case illustrates the importance of considering this diagnosis in patients who inject insulin and develop localised injection-site pain.

**Learning points:**

- Fat necrosis is a rare complication of insulin injections that can manifest with severe, persistent and well-localised pain.
- Fat necrosis can masquerade as other pathologies causing diagnostic confusion.
- The imaging modality of choice for accurate diagnosis of fat necrosis is MRI.
- Histological confirmation of fat necrosis is important.
- Appropriate management of localised fat necrosis is surgical excision, with avoidance of further insulin injections into the affected area.

## Background

Diabetes mellitus (DM) is very common. The prevalence of DM overall is increasing, and the number of patients with DM who inject insulin therapies is also increasing. Problems associated with insulin injections, including erythema, pruritus and lipohypertrophy, are relatively common and often mild and transient in their severity and duration respectively. However, we report a case of an extremely rare complication relating to insulin injections, and one that was associated with severe, persistent and localised pain with substantial adverse impact on lifestyle: superficial fat necrosis. Seemingly innocuous when viewed on an magnetic resonance imaging (MRI) scan (a 1 cm superficial lesion), the nodule caused the patient much distress over a prolonged period.

The importance of our case is that it raises awareness among the relevant healthcare professionals of superficial fat necrosis as a rare complication of insulin injections in patients with DM, the difficulties associated with its diagnosis and the importance of MRI scanning and prompt surgical excision as a highly effective treatment modality.

## Case presentation

The case is that of a 34-year-old lean female nursery nurse who had been followed-up in the Diabetes Clinic at WISDEM centre, UHCW. She had longstanding type 1 DM (T1DM), for which she was being treated with a basal-bolus regimen of insulin injections (once-daily Lantus injections, and Novorapid injections with each meal, three times per day). She had a good injection technique and had had no problems with her insulin therapy. She had good glycaemic control and stability. She had not developed any diabetes-related complications and her past medical history was otherwise nil of note. There were no other medications and no known allergies.

Six months ago, and a few weeks following her diabetes outpatient review at WISDEM centre, she developed sudden-onset, well-localised, persistent and severe sharp pain in the right iliac fossa, just lateral to the para-umbilical area. The pain did not radiate but was worsened by activity (such as walking) and relieved by simple analgesia. The pain was associated with nausea and occasional dizziness. There were no other symptoms relating to the gastrointestinal, urinary or reproductive systems.

On presentation to A&E, it was noted that her appendix was still *in situ*. A pelvic ultrasound scan revealed a right-sided ovarian cyst. Following discharge from A&E with simple analgesia, she had subsequent gynaecological follow-up 4 weeks later. At this time, her pain remained severe and constant, often requiring her to take time off from her work. Menstrual history revealed regular periods and no other symptoms suggestive of gynaecological pathology. On examination, she was noted to have an exquisitely tender subcutaneous nodule measuring 2 cm in diameter, co-localising to the site of her pain. An MRI scan was arranged, following which she was seen in the Diabetes Clinic.

Clinical assessment in the Diabetes Clinic did not reveal any history of abdominal wall trauma or pancreatitis. Her injection technique was observed to be good, and she confirmed that she had been rotating her insulin injection sites in her thighs and abdomen, but had been avoiding abdominal insulin injections as she developed pain. On examination, presence of a localised tender abdominal subcutaneous nodule was confirmed. There was no guarding and abdominal examination did not reveal any other abnormalities.

## Investigation

During her A&E assessment, a pregnancy test was found to be negative, and her biochemistry was normal (including full blood count, amylase and renal and liver functions). She was noted to have excellent blood glucose control with a recent HbA1c level of 47 mmol/mol. There was no indication of any infective or inflammatory pathology. A transvaginal ultrasound scan showed a 27 mm right-sided ovarian cyst, with no free fluid in the abdominal cavity. The ovarian cyst was thought to be the cause of her symptoms. Repeat ultrasound scan during subsequent gynaecological review confirmed the presence of a simple 10 mm physiological cyst on the right ovary and no other abnormalities in the region of interest.

**Figure 1:**
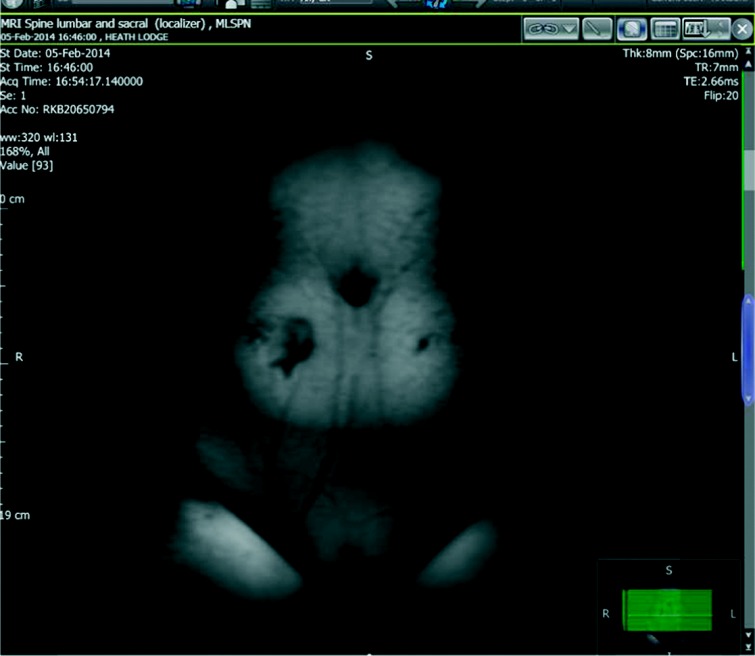
MRI image of the subcutaneous fat necrosis.

A subsequent MRI scan (Fig. 1) revealed a peri-umbilical mass in the subcutaneous fat to the right of the midline co-localising to her pain. The mass measured 1 cm in diameter, and adjacent to it there were several tiny hyperintense foci in the subcutaneous fat (to the left of the midline). The larger painful lesion on T1 imaging had characteristic features with a central fat signal surrounded by a peripheral hyperintense ring, features characteristic of fat necrosis. There was no evidence of peri-umbilical hernia, adenopathy, bowel mass or ascites. The liver, kidneys and uterus appeared normal. It was felt that the most likely cause of fat necrosis in these areas was secondary to injection of insulin.

**Figure 2:**
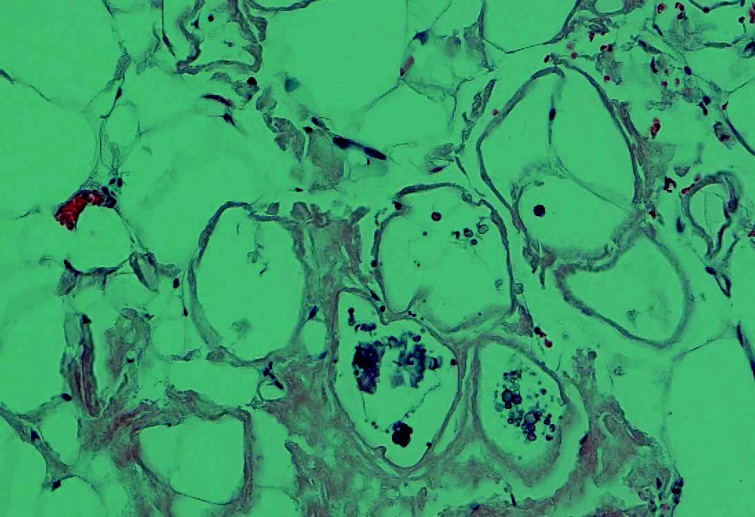
Histopathology image of the subcutaneous fat necrosis. The image specifically shows fibro-hyaline scar tissue and small amount of foreign material.

Macroscopically, histopathology of the excised lesion revealed ragged fatty tissue fragments measuring 53 mm in aggregate. Microscopically, there were small areas of fat necrosis, fibro-hyaline scar tissue, focal foreign body-type giant cells and small amount of foreign materials. Features were in keeping with the clinical diagnosis of fat necrosis (Fig. 2).

## Treatment

Following her MRI scan and diagnosis of fat necrosis, she was referred urgently for surgical opinion. She proceeded to surgical excision of the fat necrosis nodule and was discharged the following day after her operation with no complications. She maintained stable glycaemia throughout.

## Outcome and follow-up

Post-operatively, she made an excellent recovery. On further follow-up, her surgical wound has healed well. She has been pain-free since her operation, and there has been no need for her to take any further analgesia. She has not developed any further painful nodules. She continues to rotate her insulin injection sites.

## Discussion

Complications relating to insulin injections, including erythema, pruritus and indurations, are relatively common and can interfere with metabolic control (for example, through impaired absorption of insulin). Lipohypertrophy, presenting as a soft non-tender dermal nodule with associated fat cell hypertrophy, is the commonest complication relating to insulin therapy (1). Lipohypertrophy can be mitigated somewhat through rotation of insulin injection sites or use of rapidly absorbed insulin analogues. If lipohypertrophy remains troublesome despite this, there is an option of surgical removal through liposuction, although this is rarely required (1). In contrast to lipohypertrophy, fat necrosis is an extremely rare complication of insulin injections.

Fat necrosis, first described in the breast in 1920 (2), is rare. Of all its potential causes (including pancreatitis), insulin injections are among the rarest ones. The aetiology of fat necrosis is thought to result from local trauma and tissue injury caused by both physical and chemical insults, stimulating an inflammatory reaction within the adipose tissue (3). Aseptic saponification of fat by lipases was proposed to be responsible for this inflammation (4). Fat necrosis is recognised histologically as fat-filled macrophages and foreign body giant cells surrounded by interstitial infiltration of plasma cells (4). As illustrated in our case, fat necrosis is often encapsulated, which is thought to help prevent extension and spread of the inflammatory lesion into surrounding tissues (5). Fat necrosis can present at any age and usually occurs on lower extremities and breast tissue (often following an unnoticed injury) (6). One of the problems of literature searches on fat necrosis is that terminology is varied, including ‘nodular-cystic fat necrosis’, ‘mobile encapsulated lipoma’, ‘nodular fat necrosis’ and ‘post-traumatic fat degeneration’ (7).

Most case reports of fat necrosis in the literature are linked to trauma (8). In fact, we could only identify two case reports of fat necrosis that may have resulted from insulin injections. The first of these, published in 1981, was a report of a patient who developed tender subcutaneous nodules and was later diagnosed with insulin-secreting islet cell carcinoma, which may have contributed to her fat necrosis (9). The other case report was published in 2011: a case of non-encapsulated fat necrosis in a patient with T1DM who had poor glycaemic control (10).

Perhaps, owing to the apparent rarity of fat necrosis resulting from insulin injections, this possibility was not even entertained when our case first presented to A&E with abdominal pain. Instead, her pain was wrongly attributed to an ovarian cyst, illustrating the potential for superficial fat necrosis to masquerade as other pathologies, especially when affecting the abdominal subcutaneous adipose tissue. What was striking about the clinical presentation of our case though was the severity and persistence of her pain, lasting for months. Suggestive features of localised fat necrosis, illustrated by our case in her presentations following her initial A&E attendance, include extreme tenderness over the site of her pain, associated with a nodular swelling. Our case illustrates the utility of MRI used diagnostically for fat necrosis, and the importance of subsequent histopathology. Our case demonstrates the effectiveness of surgical excision in such cases, and the excellent clinical response to such management, our patient being pain-free following her operation.

Although rare, and with very few published similar case reports, it remains possible that fat necrosis resulting from insulin injections is under-recognised and under-reported. It is possible that cases with milder and more transient painful symptoms are not reported by patients. It is important though for healthcare professionals to be mindful of fat necrosis as a possible complication of insulin injections and to institute prompt and appropriate imaging investigations and surgical management wherever possible in those patients with localised and persistent superficial nodular pain and tenderness at injection sites.

## Patient consent

Informed consent was obtained from the patient for publication of this (anonymised) submitted case report and accompanying images.

## Author contribution statement

I confirm that all co-authors listed contributed substantially to the preparation of this manuscript. The corresponding author (Dr T M Barber) is the named physician of the patient.
